# *E. coli* mutant NR71 grows on succinate in the absence of an F_1_ ATPase

**DOI:** 10.1128/jb.00171-26

**Published:** 2026-09-14

**Authors:** Wiliam S. A. Brusilow

**Affiliations:** 1 Department of Biochemistry, Microbiology, and Immunology, Wayne State University School of Medicine 12267, Detroit, Michigan, USA; Dartmouth College Geisel School of Medicine, Hanover, New Hampshire, USA

**Keywords:** *E. coli* ATPase mutant, oxidative phosphorylation

## Abstract

Over 50 years ago, two *E. coli* ATPase mutants were isolated and characterized. The first, NR70, was later shown to contain a 21-base deletion in the gene for the gamma subunit of the F_1_F_0_ ATPase, rendering it incapable of growth on the nonfermentable carbon source succinate. The second, NR71, was derived from NR70 by selection for growth on succinate. We show that NR71 contains a stop codon near the middle of the gene for the alpha subunit, making it unable to synthesize a full-length alpha subunit. We further show that we could not detect the alpha, beta, or gamma subunits in membranes isolated from this strain. Strain NR71 can grow on succinate despite the absence of an intact F_1_ ATPase and therefore carry out succinate-dependent ATP synthesis by an unknown process.

**IMPORTANCE:**

An *E. coli* strain lacking an ATPase can nonetheless grow on a non-fermentable carbon source, indicating that this mutant has an unknown mechanism for synthesizing ATP from the oxidation of succinate.

## INTRODUCTION

Most ATPs are synthesized by phosphorylation coupled to the redox reactions of electron transport. The oxidation of sugars, fats, and the carbon skeletons of amino acids is used to reduce cofactors such as NAD and quinones, which then participate in a series of membrane-bound redox reactions, where the energy released in those processes is coupled to the formation of a transmembrane chemical and electrical gradient, typically a proton motive force. The enzyme that converts the proton motive force to ATP is the F_1_F_o_ ATPase/ATP synthase, a large membrane-bound complex that, in facultative bacteria that can grow aerobically or anaerobically, can either use the proton motive force to synthesize ATP or use ATP to create a transmembrane electrochemical gradient of protons.

The F_1_F_o_ ATPase consists of the membrane-bound F_o_ sector (three subunits: a, b, and c), which acts as a transmembrane proton channel, and an extrinsic F_1_ sector (five subunits: α, β, γ, δ, and ε) that contains the catalytic sites for ATP synthesis or hydrolysis. This enzyme has been characterized in many organisms, and all have this same basic two-sector structure, although species-specific differences exist in the number of subunits in each sector (1–3).

Historically, bioenergetic studies of ATP synthesis were conducted primarily in mitochondria and chloroplasts, but around 1970, because of the ability to apply genetic approaches to bacteria, several laboratories began to study the F_1_F_o_ ATPase in *E. coli* by isolating ATPase mutants and characterizing the effects of those mutations on the various activities of the enzyme, including ATPase activity, the coupling of ATPase activity to the formation of a proton motive force, and the ability of the F_o_ to conduct protons in the absence of the F_1_ (4–7).

One of the ATPase mutants (then designated *unc* mutants for uncoupled, now referred to as *atp* mutants) isolated during that period was strain NR70 (5), which was able to grow aerobically on glucose but did not grow aerobically on the nonfermentable carbon source succinate, and which was found to contain an F_o_ sector that appeared to be more permeable to protons than the F_o_ of the wild-type parental strain.

Subsequent analyses of strain NR70 showed that this mutant carried a 21-base-pair deletion in the *atpG* gene that resulted in a 7-amino-acid deletion within the F_1_ gamma subunit, and that (i) eliminated growth on succinate, (ii) eliminated ATPase activity, and (iii) resulted in a more proton-permeable F_o_ sector (8, 9).

The same laboratory then attempted to isolate revertants or suppressors of NR70, strains that had regained the ability to grow on succinate. They characterized one such mutant, strain NR71. Their characterization of this second mutant, which turned out to be a suppressor, demonstrated that both NR70 and NR71 membranes contained F_o_ subunits that could effectively bind F_1_ and carry out ATP-driven proton pumping. Unlike the membranes from NR70, membranes from NR71 were no more proton permeable than wild type, so they concluded that this second mutation was in the F_o_ sector (10).

The studies in this paper were initiated by the question of how an ATPase mutant might change from Succ^−^ to Succ^+^ when that mutant carries a 21-nucleotide deletion in one of the F_1_ genes. These studies characterize the second mutation and show that this strain is a suppressor of the original phenotype that grows on succinate without an F_1_ ATPase.

## MATERIALS AND METHODS

*E. coli* strains 7 (the starting strain, a strain that produces an α-glycerophosphate dehydrogenase constitutively (i.e., not requiring induction), NR70, and NR71, and the methods by which they were selected, have been described previously (5, 10, 11).

Cells were grown on rich medium (Luria Broth—10 g/L Bacto tryptone, 10 g/L NaCl, and 5 g/L Bacto yeast extract) to an OD_650 nm_ of 0.4–0.6. Membranes were isolated and assayed for protein, ATPase, ATP synthase, and NADH-driven fluorescence quenching as described previously (12–16).

Minimal medium consisted of 50 mM KPi, pH 7.1, 1 g/L (NH_4_)_2_SO_4_, 10 mg/L thiamine, and 10 mM MgCl_2_. Succinate or glucose was added to liquid media and 1.5% agar plates to a final concentration of 4 g/L. Our stock trace metal solution contains the following (all concentrations in millimolar): 0.5 ZnSo_4_, 0.5 CuSO_4_, 1.0 MnSO_4_, 0.004 Na_2_MoO_4_, 0.005 CoCl_2_, 1.0 FeSO_4_, 2.0CaCl_2_, 100 KCl, and 100 NaCl. This solution was diluted 1,000-fold into all minimal media. Growth curves were determined for cells grown in 50 mL of medium in 500 mL shake flasks shaken at 34°C at approximately 120 rpm.

Genomic DNA sequencing was carried out in the sequencing facility at Michigan State University.

The *E. coli* F_1_ ATPase was purified according to Foster and Fillingame (17).

### Membrane proteins were separated on SDS acrylamide gels and visualized as follows

Approximately 10 μg of protein were loaded in each lane of a 12.5% acrylamide Laemmli SDS gel (18). Following electrophoresis, separated proteins were transferred to nitrocellulose by electrophoresis at 100 volts for 30 min in 25 mM Tris base, 192 mM glycine, and 20% (vol/vol) methanol transfer buffer pH 8.3; blots were treated with an antibody to either the alpha subunit (cross-reacting with the beta subunit), the gamma subunit, or the c subunit, followed by treatment with a secondary antibody conjugated to alkaline phosphatase (Sigma Aldrich A2556) and developed with a BCIP/NBT substrate (Sigma Aldrich B1911). To purify anti-alpha, we followed the process described in Fang (19). The subunits in purified F_1_ were separated on an SDS gel followed by transfer to nitrocellulose as mentioned above. The subunits were visualized on nitrocellulose with ruthenium red, and the band corresponding to alpha was cut out and incubated overnight with anti-F_1_ to bind the antibodies specific to the α subunit. Those antibodies were eluted from the nitrocellulose, washed, and dialyzed against phosphate-buffered saline pH 7.4 (10 mM phosphate, 137 mM sodium chloride, and 2.7 mM potassium chloride), then used in the blot described in Fig. 4. Antibodies specific to the c subunits were purchased from Quality Controlled Biochemicals, Inc., Hopkinton, MA 01748, and antibodies specific to the gamma subunit from Thermo Fisher Scientific, Rockford, IL 61105. These were raised by injecting rabbits with peptides specific for each subunit, resulting in subunit-specific antiserum.

### P1 transductions were carried out as follows

Strain 7 was made ilv^−^ by P1 transduction of the TN10::ilv from LE392Tn10::ilv into strain 7. The transduction was verified by the conversion of strain 7 from Ilv^+^ to Ilv^−^ and from tetracycline-sensitive to tetracycline-resistant. The Tet-resistant ilv^−^ strain 7 was then transduced to Tet-sensitive ilv^+^ with a P1 lysate made on strain NR71 and plated on minimal succinate plates lacking ilv. Since all cells were Succ^+^, this selection was for the isoleucine-valine locus that contransduces with the atp locus with a frequency of 50% (4, 6). The resultant ilv^+^ colonies were observed in comparable numbers for bigger (growing well) and smaller (growing substantially less well) colonies, as would be expected for a 50% transduction rate. To both verify the products of this transduction and minimize the small possibility of selecting for a different Succ^+^ mutation, chromosomal DNA was isolated from four smaller Succ^+^Tet-sensitive colonies, and the *atp*A-*atp*G region containing both mutant sequences found in NR71 was PCR-amplified and sequenced. All four contained *both* the deletion at the start of *atp*G and the same *atp*A nonsense mutation identified in chromosomal sequencing of NR71.

ATP levels in cells were measured by growing cells on the minimal media containing either glucose or succinate to OD_650 nm_ of 0.4–0.6, at which point samples were removed. For protein determinations, cells were TCA-precipitated and the precipitates assayed for protein using the Lowry assay (20). For ATP determinations, samples were chilled in ice-cold water and centrifuged as quickly as possible. The pellets were resuspended in 6% perchloric acid, and ATP levels were assayed using the luciferase assay, as described previously (16).

## RESULTS

**Fig 1:**
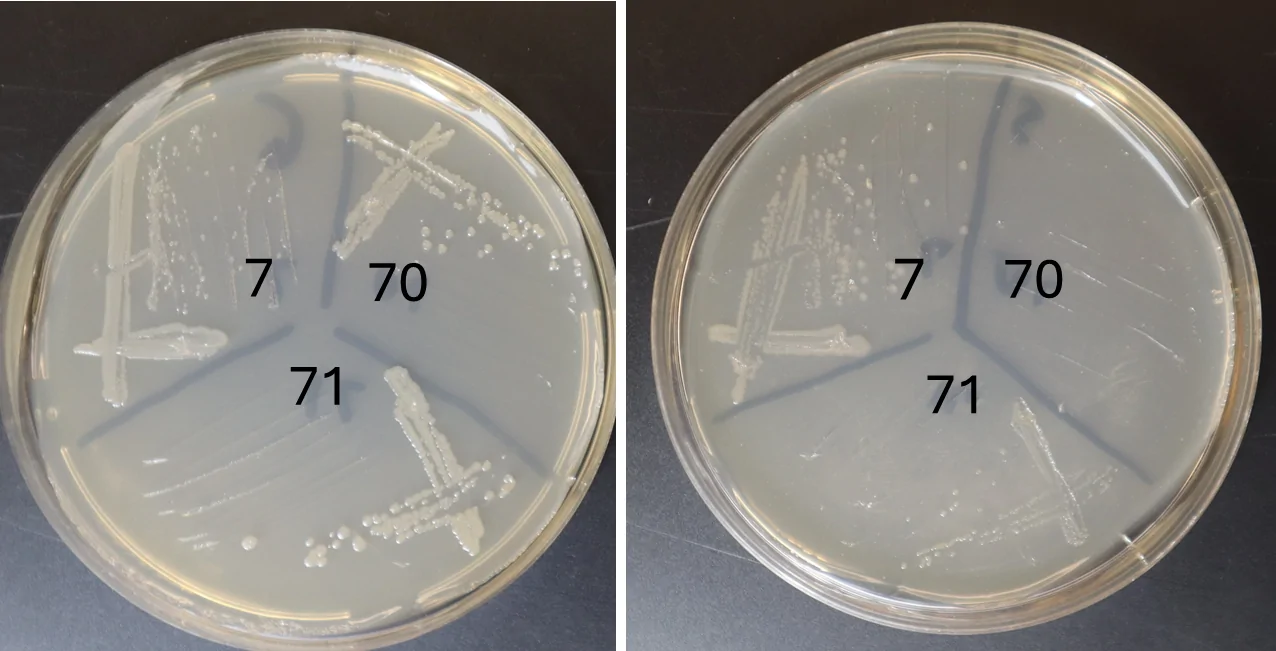
Growth on minimal media at 37°C. Strains 7, NR70, and NR71 were streaked on minimal plates containing either 0.4% glucose or 0.4% succinate as the sole carbon and energy source. Left (glucose minimal medium): strains 70 and 71 were streaked out and incubated at 37°C for 2 days, at which time strain 7 was streaked out and the plate incubated for another 2 days. Right (succinate minimal medium): strains NR70 and NR71 were streaked out and incubated at 37°C for 4 days, at which time strain 7 was streaked out and the plate incubated for an additional 2 days at 37°C.

**Fig 2:**
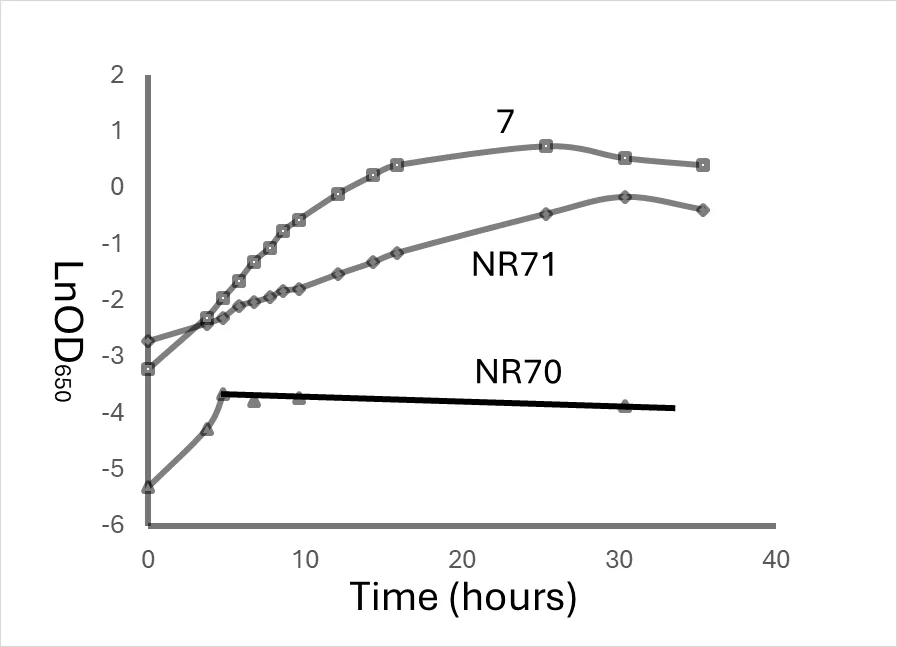
Growth of strain 7, NR70, and strain NR71 on minimal succinate medium. After overnight growth on minimal glucose medium, each strain was transferred (5% inoculum) to minimal succinate medium at 34°C. Culture turbidity was measured as the optical density at 650 nanometers (OD_650 nm_).

NR70 was one of the earliest mutants isolated in the *E. coli* ATPase (5). Lacking a functional ATPase/ATP synthase, NR70, like all such *unc/atp* mutants, cannot grow on a nonfermentable carbon source such as succinate or acetate/malate. That same lab isolated a revertant of NR70 that could grow on succinate and designated that strain NR71 (10). We started our studies by confirming that observation, as shown in Fig. 1 and 2. Clearly, NR70 does not grow on minimal succinate, and NR71 does, as seen in the original study. We further characterized this growth by assaying cellular ATP levels in whole cells of these three strains grown on minimal glucose medium and for strains 7 and NR71 grown on succinate.

### Growth of strains 7 and NR71 on liquid medium

Figure 2 shows the growth curves for strain 7 and strain NR71 on minimal medium containing 1% succinate. The doubling time for strain 7 under these conditions was approximately 2 h, and for strain NR71 approximately 5 h. The final OD for strain NR71 was approximately half of that for strain 7. Strain NR70 does not grow on succinate (after using up the glucose present in the inoculum).

Table 1 shows the ATP content of cells grown as described in Materials and Methods. There was essentially no difference in ATP content of these strains when grown on minimal glucose medium, but strain NR71 contained only 10% of the ATP in strain 7 when both were grown on minimal succinate medium. No values are shown for NR70 in minimal succinate because it does not grow on that carbon source.

**TABLE 1:** ATP content of cells grown on minimal media^*a*^^,^^*b*^

ATP content (μmole/g protein)					
	Minimal glucose medium			Minimal succinate medium	
Strain	7	NR70	NR71	7	NR71
Avg.	3.6	3.1	3.7	5.3	0.58
S.E.M.	0.9	1.9	0.9	1.2	0.13
*N*	4	3	5	5	4

^*a*^ ATP content given in µmole/g protein.

^*b*^ *N,* number of independent cultures assayed; S.E.M., standard error of the mean.

### Identification of the mutation in NR71

**Fig 3:**
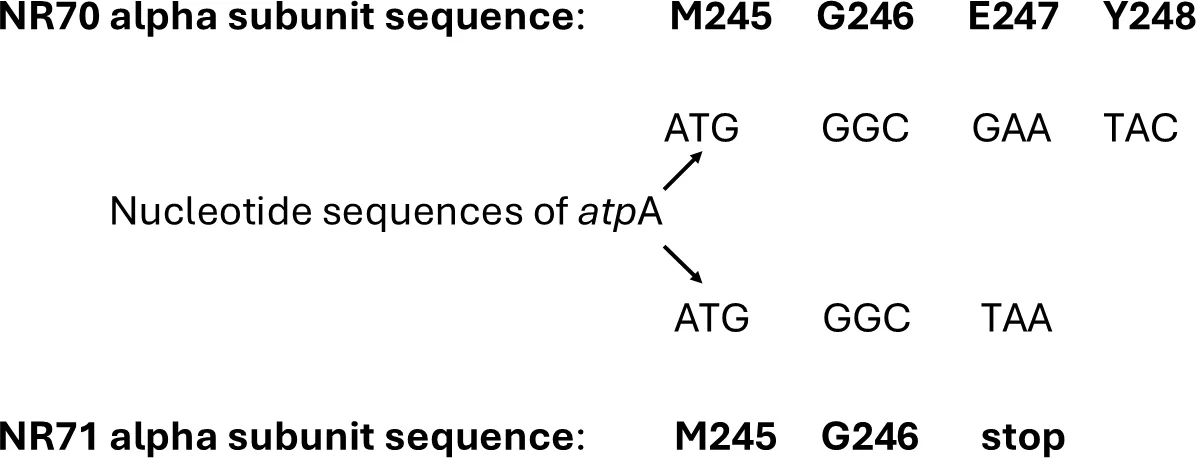
A G → T mutation in *atp*A of NR71 creates a stop codon near the middle of the gene for the ATPase alpha subunit.

To determine the nature of this mutation, the entire genome of NR71 was sequenced and compared to the genome sequence of NR70. The genomic sequencing showed that, in addition to the 21-base deletion from the parent strain NR70, NR71 has an additional mutation near the middle of *atp*A, the gene for the alpha subunit of the F_1_. A C → A mutation at *E. coli* genomic position 3,919,119 (NCBI Reference Sequence NC_000913.3), shown in the figure as the G → T mutation in the opposite strand, produces a stop codon that replaces glutamate 247 of that subunit (Fig. 3). NR71 therefore cannot make a complete alpha subunit.

The genomic sequencing discovered one additional mutation unique to strain NR71, a G → A at position 3,980,479 (NCBI Reference Sequence NC_000913.2); this is an intergenic region. If we assume no differences in the region between the two mutations, the position of this second mutation in the updated NCBI Reference Sequence NC_000913.3 would be 3,982,456.

Upon receiving these results, we PCR-amplified the *atp*A gene from both strains and had them sequenced at the Wayne State sequencing facility, which confirmed the presence of the *atp*A mutation found in NR71.

To show that the phenotype conversion was the result of this mutation and not some change elsewhere in the chromosome of NR71, we used P1 transduction to move the *atp* genes from NR71 back into strain 7 (see Materials and Methods), which showed that the Succ^+^ phenotype was conferred by this region of the chromosome and specifically by these two mutations.

**Fig 4:**
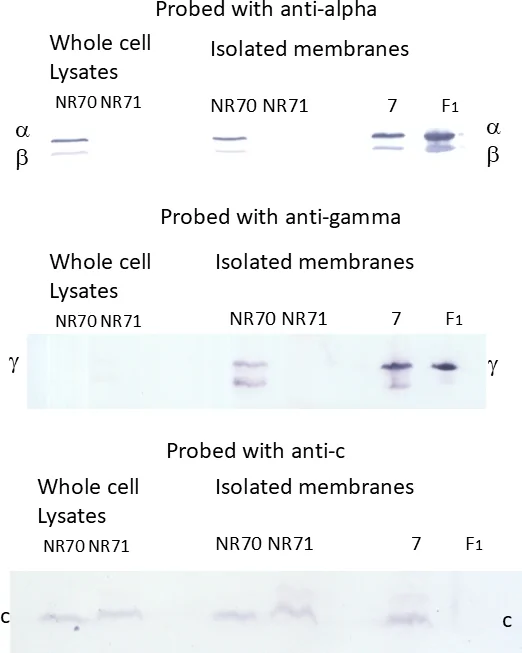
Immunoblots of membranes and whole-cell lysates from strains 7, NR70, and NR71. Approximately 10 μg of protein was loaded in each lane. After transfer to nitrocellulose, blots were treated with an antibody to either the alpha subunit (cross-reacting with the beta subunit), the gamma subunit, or the c subunit, followed by treatment with a secondary antibody conjugated to alkaline phosphatase and developed with a BCIP/NBT substrate. The locations of the alpha (α), beta (β), gamma (γ), and c ATPase subunits are indicated on both sides of the blots.

To further characterize the effects of this mutation, we conducted immunoblot analysis of whole-cell lysates and isolated membranes prepared from strains 7, NR70, and NR71 using antibodies specific for the alpha, gamma, and c subunits. Not surprisingly, the antibody to the alpha subunit cross-reacts with the beta subunit, since these two subunits are homologous (21). Figure 4 shows that membranes from strains 7 and NR70 both contain the alpha, beta, and gamma subunits of the F_1_ as well as the c subunit of the F_o_. The alpha and beta subunits are also seen in the whole-cell lysates, but although the gamma subunit was clearly seen in the membranes, it was apparently not present in high enough concentration to be visualized in the immunoblots of whole-cell lysates. None of these F_1_ subunits are found in the membranes of strain NR71, although in both the crude lysate and purified membranes, the c subunit is present in amounts comparable to those found in strains 7 and NR70. Immunodiffusion experiments in the original characterization of NR71 suggested that this strain did make appreciable amounts of the beta subunit (8). Our studies, however, appear to indicate that it does not.

**Fig 5:**
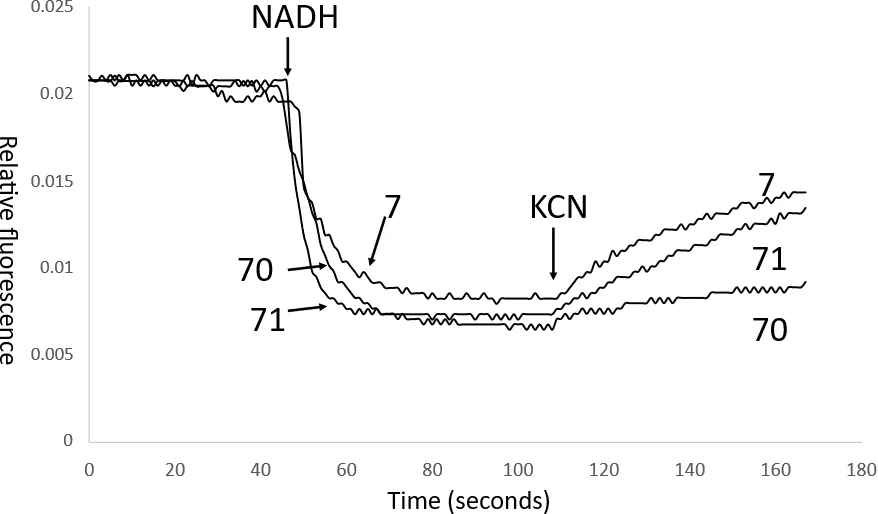
Relative membrane energization by NADH-driven electron transport in membranes isolated from strains 7, NR70, and NR71, measured by the quenching of fluorescence from the fluorophore ACMA. Reactions were conducted in 2 mL of buffer containing 0.25 mg of membrane protein. NADH (0.6 mM) and KCN (1 mM) were added at the indicated times.

We assayed membranes from these three strains for ATPase and ATP synthase activity. The results are shown in Table 2. Neither of the mutant strains contains significant amounts of these activities. This result is not surprising, since NR70 contains a defective ATPase, and NR71 does not contain an F_1_ ATPase. A few experimental preparations from NR71 appeared to have low ATPase activities that are higher than those found in preparations from NR70, but only slightly, and the standard error for these measurements is high enough to make the significance of this difference questionable. The ATP synthesis assay of isolated membranes appears to have a background of approximately 20 nmoles/min/mg. We do not know the basis for this background, but the low activities measured in the NR70 and NR71 membranes are not significantly different from those measured in the strain 7 membranes in the presence of the uncoupler CCCP. Furthermore, we measured NADH-driven fluorescence quenching by these membranes in the presence of the fluorophore ACMA. This assay provides a qualitative measure of how well the electron transport chain can create a transmembrane electrochemical gradient of protons. Figure 5 shows that membranes from all three strains are capable of carrying out proton translocation coupled to oxidation of NADH. All three membrane preparations produced comparable fluorescence quenching. It should be noted that the small leak seen when right-side-out vesicles made from NR70 are energized by D-lactate is not seen in these everted membrane vesicles energized by NADH, which has been shown to drive respiration almost 10 times more strongly than D-lactate (5).

**TABLE 2:** ATPase and ATP synthase activities in membranes^*a*^^,^^*b*^

ATPase activity				ATP synthase activity					
Strain	7	NR70	NR71	7	7 + CCCP	NR70	NR70 + CCCP	NR71	NR71 + CCCP
Avg.	1.4	0.015	0.14	371	28	14	20	40	25
S.E.M	0.085	0.005	0.11	24	7.3	2	3.8	16	4.3
N	4	4	4	4	4	3	3	4	4

^*a*^ Membranes were prepared and assayed as described in Materials and Methods. CCCP was added to a final concentration of 100 µM. ATPase activity (phosphate production) is given in micromoles/min/mg of membrane protein. ATP synthase activity (ATP produced) is given in nanomoles/min/mg of membrane protein.

^*b*^ *N,* number of independent cultures assayed; S.E.M., standard error of the mean.

## DISCUSSION

ATP synthesis can result from either substrate-level phosphorylation or oxidative (electron-transport-driven) phosphorylation. Substrate-level phosphorylation involves the formation of a molecule containing a so-called “high-energy phosphate,” which can then be transferred to ADP to form ATP. Examples in the glycolytic pathway are the phosphoglycerate kinase reaction and the pyruvate kinase reaction. Oxidative phosphorylation does not involve a high-energy phosphorylated intermediate, but rather couples the redox reactions of the electron transport chain to the creation of a transmembrane electrochemical gradient of protons, also called the proton motive force, followed by the coupling of that proton motive force to ATP synthesis by the F_1_F_o_ ATPase/ATP synthase.

Nonfermentable carbon sources like succinate, which cannot enter the glycolytic pathway leading to either of the substrate-level phosphorylation reactions of that pathway or any other pathway involving high-energy phosphorylated compounds, can generate ATP only by oxidative phosphorylation for *E. coli*; therefore, growth on succinate or any other non-fermentable carbon source is a test for the presence of a functional F_1_F_o_, as described in the earliest studies on the isolation and study of such mutants (4–7). The complete oxidation of succinate to four molecules of CO_2_ can produce multiple reduced FADH_2_ and NADH cofactors and one molecule of ATP by the reactions of the TCA cycle, but this single ATP is inadequate to support growth in the absence of oxidative phosphorylation. In our previous experience, we have found no ATPase mutant that grows on minimal succinate medium.

For NR71, however, this is not the case. The strain from which NR71 was derived, the neomycin-resistant NR70, contains a 21-base deletion in the gene for the gamma subunit. This strain therefore makes a gamma subunit missing seven amino acids near the N-terminus, which produces a Succ**^−^** phenotype. Starting with NR70, NR71 was selected for its ability to regain growth on succinate (10), and our study shows the restoration of growth on succinate is associated with a nonsense mutation located about halfway through the gene for the alpha subunit.

These results raise the obvious question of whether the partial alpha subunit might still confer, or in this case restore, functionality to a defective ATPase. The predicted product of the alpha gene in NR71 would have a molecular mass of 25,891. We could not detect such a truncated alpha in immunoblots, nor could we detect the beta subunit, so such a modified F_1_ ATPase appears to be present at very low levels in membranes, if at all. Also, the catalytic site for ATP synthesis and hydrolysis has been described for the bovine ATPase as consisting of amino acid residues from both the alpha and beta subunits (22). Of the 16 residues of the alpha subunit found in the catalytic site of the bovine ATPase, 15 are identical to those residues in the alpha subunit of the *E. coli* ATPase (23). The truncated alpha subunit would contain six of those (with the bovine S177 replaced by *E. coli* A177), including the Walker A sequence (24). However, 10 of the alpha subunit residues found in the catalytic site would be missing and therefore severely inhibiting or preventing the formation of a correct and functional catalytic site. Even if such assembly occurred, the complex would still contain the original mutant gamma subunit in NR70. We therefore feel it unlikely that the truncated alpha subunit made from the prematurely terminated *atpA* gene could be assembled into an active ATPase that would effectively suppress or cure the *atpG* mutation found in NR70.

We did not assay transcription of the individual genes in any of these strains to determine whether the stop codon in the middle of the atpA gene might be exerting polarity over the downstream genes for the gamma and beta subunits, and thus affecting their expression and therefore accounting for the absence of the gamma and beta subunits in our immunoblots.

The original report describing NR71 showed that membranes of both NR70 and NR71 could be reconstituted with F_1_, and those reconstituted membranes demonstrated ATP-driven proton pumping (10), indicating that both strains contained F_o_ subunits capable of binding F_1_ sectors. Our immunoblots indicated the presence of the F_o_ c subunit in their membranes. In those original studies, the F_o_ subunits in NR71 membranes, despite being able to have proton-conducting function restored by exposure to F1, did not appear to be very permeable to protons compared to the F_o_ sector in membranes isolated from strain NR70, and that study therefore concluded, understandably but mistakenly, that the NR71 mutation was in the F_o_.

It has since been demonstrated that during synthesis and assembly, the F_o_ subunits exist in some arrangement that conducts protons poorly in the absence of the F1 and not as readily as the F_o_ in the fully assembled F_1_F_o_ complex. This “closed” F_o_ is then converted to its mature proton-conducting form by interactions with F_1_ subunits during assembly (12–14). It appears that the NR71 mutation in atpA might interfere with this maturation step, so the F_o_ subunits are present in the membrane but conduct protons poorly. It has been shown that such an immature F_o_ can still bind F_1_ and carry out ATP-dependent energy transduction (12, 14).

How does NR71 synthesize ATP from succinate without an F_1_ ATPase? These studies describe the mutation in NR71 and its effect on cellular ATP levels and ATPase activities, and therefore do not directly address this question, so we do not know and can only speculate.

As argued above, it is unlikely that a combination of a mutant gamma subunit and a truncated alpha subunit missing most of the amino acids found in the catalytic site might assemble into a functional ATP synthase, especially since NR71 cells do not appear to produce detectable amounts of beta, gamma, or truncated alpha in our experiments, leading to the conclusion that strain NR71 appears to contain another mechanism for oxidative phosphorylation that becomes apparent when the major energy coupling complex of the F_1_F_o_ ATPase is absent, and that the elimination of the F_1_ sector in NR71 might somehow unmask that activity. This second theory, however, fails to explain how such growth is not seen in strains containing large or complete deletions of the *atp* operon, although NR71 does contain intact genes for the F_o_ sector and the delta subunit. The results therefore raise the possibility that this activity is unique to NR71.

We have not looked at other strains of *E. coli* carrying the same combination of mutations as exist in NR71. The starting strain 7 for these studies was created in 1964 through multiple steps of selection and screening (11). The observed phenotype in NR71 might therefore involve strain-specific additional mutations that arose during that process but were not characterized, but we have not investigated this possibility.

Finally, in P-31 NMR studies of phosphorylated compounds in living *E. coli,* it has been reported that NR71 cells grown on minimal glucose medium contain significant amounts of a phosphodiester compound that is not seen in NR70 (25). This compound was not characterized, but its existence does suggest that NR71 has a novel phosphorylation reaction or pathway.
